# Supplementary material for: Antibody evasion and receptor binding of SARS-CoV-2 LP.8.1.1, NB.1.8.1, XFG, and related subvariants
Source: Cell Rep. Author manuscript; Available in PMC 2025 Dec 23. (PMC12723667; doi:10.1016/j.celrep.2025.116440)
Supplement: 1 [file NIHMS2120118-supplement-1.pdf]

Cell Reports, Volume *44*

Supplemental information

Antibody evasion and receptor binding of SARS-CoV-2 LP.8.1.1, NB.1.8.1, XFG, and related subvariants

Ian A. Mellis, Madeline Wu, Hsiang Hong, Chih-Chen Tzang, Anthony Bowen, Qian Wang, Carmen Gherasim, Virginia M. Pierce, Jayesh G. Shah, Lawrence J. Purpura, Michael T. Yin, Aubree Gordon, Yicheng Guo, and David D. Ho

Supplementary Appendix

Supplementary Figures and Tables **2**
Figure S1: Phylogenetic relationships of JN.1 subvariants. 2
Figure S2: Durability of serum neutralizing titers in KP.2 MV recipients. 3
Table S1: Summary of clinical cohorts. 4
Table S2: Participant demographic, vaccine, and infection details. 5

Supplementary Figures and Tables

Figure S1: Phylogenetic relationships of JN.1 subvariants.

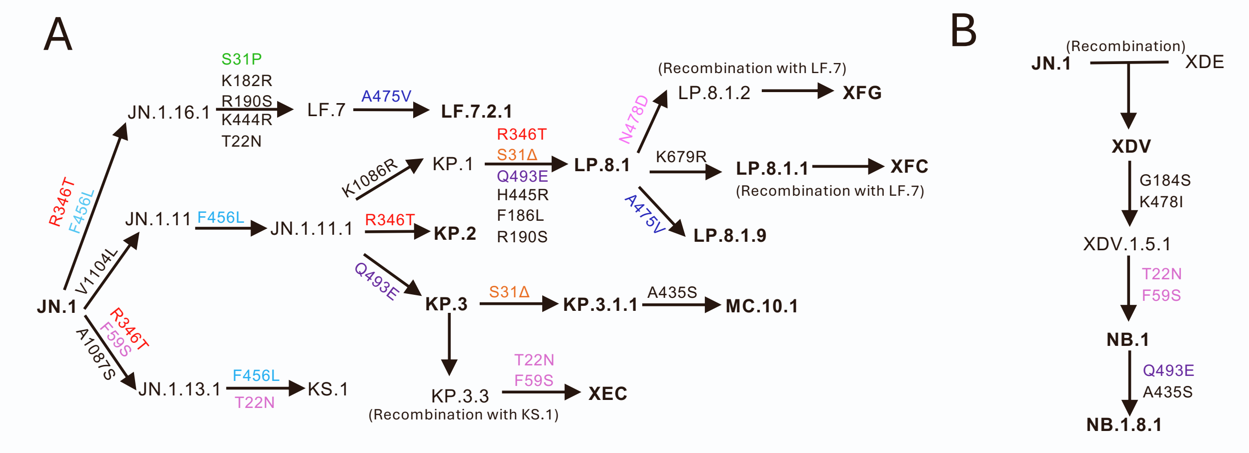

Figure S1: Phylogenetic relationships of JN.1 subvariants. Spike mutations appearing in descendants noted above each arrow. A: Phylogeny of the JN.1 sublineage, excluding NB.1.8.1. B: Recombination event and select ancestors leading to NB.1.8.1.

Figure S2: Durability of serum neutralizing titers in KP.2 MV recipients.

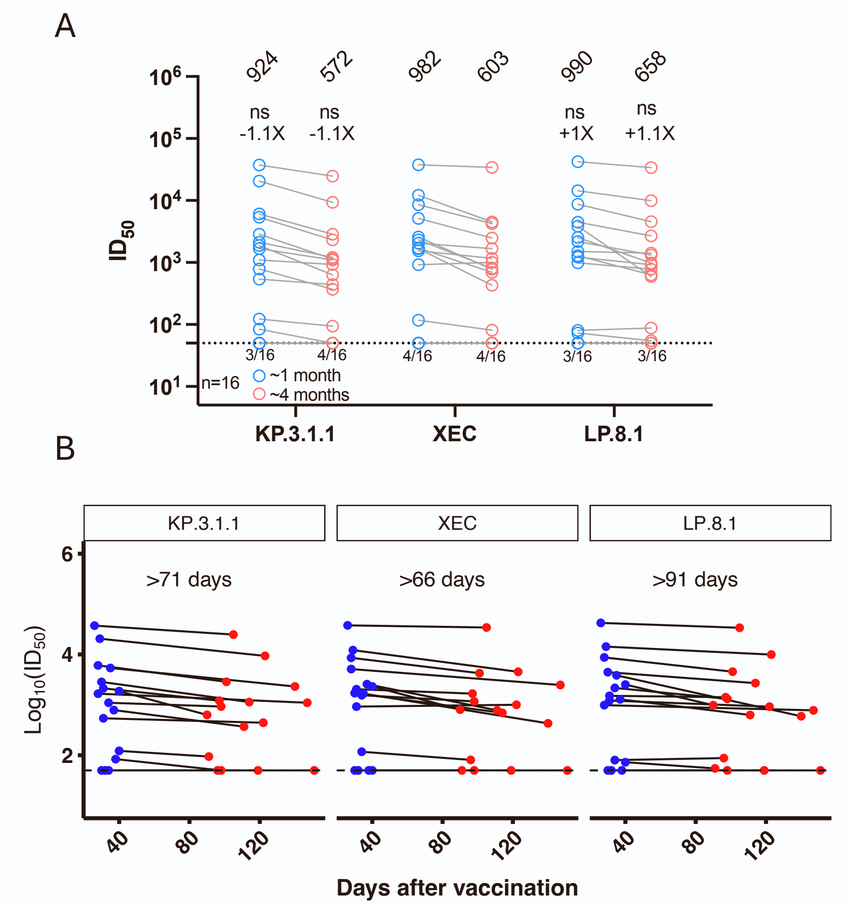

Figure S2: Durability of serum neutralizing antibody titers in KP.2 MV recipients. A: Serum neutralizing titers (ID_{50}) against VSV-based pseudoviruses bearing spike proteins of KP.3.1.1, XEC, or LP.8.1, for samples from recipients of KP.2 MV boosters at ~1 month and ~4 months time points post-booster. The geometric mean titer (GMT) is presented at the top. The fold change in GMT for each virus compared to XEC is also shown immediately above the symbols. Statistical analyses used Wilcoxon matched-pairs signed-rank tests, comparing to XEC. n, sample size; ns, not significant. * $p < 0.05$, ** $p < 0.01$, *** $p < 0.001$, **** $p < 0.0001$. Numbers under the dotted lines denote numbers of serum samples that were under the limit of detection (ID50 < 50). B: Paired ID_{50} values per participant. Geometric mean estimated half-life of titers noted for each variant. Half-life was not estimated for participants with unchanged ID_{50} (i.e., both values below LOD), nor for the one participant who had an apparent small increase in titer. Blue = first sampling post-boost, Red = second sampling post-boost.

Table S1: Summary of clinical cohorts.

		All participants		KP.2 MV		No KP.2 MV	
		No. or Mean	% or (range)	No. or Mean	% or (range)	No. or Mean	% or (range)
**Total**		40	-	20	-	20	-
**Female**		29	72.5%	14	70.0%	15	75.0%
**Male**		11	27.5%	6	30.0%	5	25.0%
**Age**		44.5	(20, 80)	42.5	(21, 80)	46.5	(20, 68)
**No. Vaccines**	All vaccines	4.6	(4, 8)	5.7	(4, 8)	3.5	(6, 8)
	WT	2.7	(2, 4)	3.1	(2, 4)	2.3	(2, 4)
	BA.5 BV	0.7	(0, 1)	0.8	(0, 1)	0.6	(0, 1)
	XBB.1.5	0.7	(0, 2)	1.0	(0, 2)	0.6	(0, 2)
	KP. 2 MV	0.5	(1,1)	1.0	(1,1)	-	-
**Sera Days Post Most Recent Infection**		525.6	(0, 1685)	672.8	(0, 1685)	404.5	(0, 1427)
**Sera Days Post Most Recent Vaccination**		302.5	(26, 1208)	33.6	(26, 50)	618.8	(26, 1208)

Table S2: Participant demographic, vaccine, and infection details.
Vaccine formulations are denoted as Wild-type (WT), BA.5 Bivalent (BA.5), XBB.1.5 monovalent (XBB.1.5), and KP.2 monovalent (KP.2). Vaccine manufacturers are denoted as Pfizer (P) or Moderna (M).

ID	Group	Age (Yr)	Sex	Race	No. Vax	No. WT Vax	No. BA.5 Bivalent Vax	No. XBB.1.5 Vax	No. KP.2 MV	Sera Days Post Most Recent Infx	Sera Days Post Most Recent Vaccine	Vaccine History
CUMC 1	KP.2 MV	25	F	As	6	3	1	1	1	880	31	WT-P/WT-P/WT-P/BA.5-M/XBB.1.5-M/KP.2-M
CUMC 2	KP.2 MV	35	M	As	4	3	0	0	1	-	26	WT-P/WT-P/WT-P/KP.2-M
CUMC 3	KP.2 MV	21	M	As	4	3	0	0	1	195	37	WT-P/WT-P/WT-P/KP.2-P
CUMC 4	KP.2 MV	29	F	As	4	3	0	0	1	244	28	WT-P/WT-P/WT-P/KP.2-M
CUMC 5	KP.2 MV	55	M	As	5	3	1	0	1	451	30	WT-M/WT-M/WT-M/BA.5-P/XBB.1.5-P/KP.2-P
CUMC 6	KP.2 MV	36	M	Wh	6	3	1	1	1	796	30	WT-P/WT-P/WT-P/WT-P/BA.5-P/XBB.1.5-P/KP.2-M
CUMC 8	KP.2 MV	23	F	As	4	3	0	0	1	-	40	WT-P/WT-P/WT-P/KP.2-M
CUMC 9	KP.2 MV	42	M	As	6	3	1	1	1	650	34	WT-P/WT-P/BA.5-P/XBB.1.5-P/KP.2-M
CUMC 11	KP.2 MV	61	F	Wh	5	3	1	0	1	826	34	WT-P/WT-P/WT-P/BA.5-P/KP.2-P
UMICH 1	KP.2 MV	29	F	Af	6	3	1	1	1	784	31	WT-P/WT-P/WT-P/BA.5-P/XBB.1.5-M/KP.2-P
UMICH 2	KP.2 MV	24	F	Wh	6	3	1	1	1	658	28	WT-P/WT-P/WT-P/BA.5-P/XBB.1.5-M/KP.2-P
UMICH 3	KP.2 MV	25	F	Wh	6	3	1	1	1	-	32	WT-P/WT-P/WT-M/BA-M/XBB.1.5-M/KP.2-P
UMICH 4	KP.2 MV	80	F	Wh	8	4	1	2	1	-	40	WT-P/WT-P/WT-P/WT-P/BA.5-P/XBB.1.5-P/XBB.1.5-P/KP.2-P
UMICH 5	KP.2 MV	33	F	As	6	3	1	1	1	-	38	WT-P/WT-P/WT-P/BA.5-M/XBB.1.5-P/KP.2-P
UMICH 6	KP.2 MV	55	F	Wh	6	3	1	1	1	653	29	WT-M/WT-M/WT-M/BA.5-M/XBB.1.5-M/KP.2-M
UMICH 7	KP.2 MV	59	F	Wh	7	4	1	1	1	-	35	WT-P/WT-P/WT-P/WT-P/BA.5-P/XBB.1.5-M/KP.2-P
UMICH 8	KP.2 MV	64	M	Wh	6	3	1	1	1	741	31	WT-P/WT-P/WT-P/BA.5-P/XBB.1.5-P/KP.2-P
UMICH 9	KP.2 MV	66	F	Wh	6	3	1	1	1	1685	31	WT-M/WT-M/WT-P/BA.5-P/XBB.1.5-P/KP.2-P
UMICH 10	KP.2 MV	27	F	Wh	5	2	1	1	1	476	37	WT-J/WT-M/BA.5-M/XBB.1.5-M/KP.2-M
UMICH 11	KP.2 MV	61	F	Wh	7	4	1	1	1	380	50	WT-P/WT-P/WT-P/WT-P/BA.5-P/XBB.1.5-P/KP.2-M
UMICH 12	No KP.2 MV	41	F	Wh	2	2	0	0	0	142	1208	WT-P/WT-P
UMICH 13	No KP.2 MV	72	F	Wh	6	3	1	2	0	109	139	WT-P/WT-P/WT-P/BA.5-P/XBB.1.5-P/XBB.1.5-P
UMICH 14	No KP.2 MV	60	F	Wh	5	3	1	1	0	573	207	WT-M/WT-M/WT-M/BA.5-M/XBB.1.5-M/XBB.1.5-M
UMICH 15	No KP.2 MV	68	F	Wh	5	3	1	1	0	785	298	WT-M/WT-M/WT-M/BA.5-M/XBB.1.5-M/XBB.1.5-M
UMICH 16	No KP.2 MV	40	F	Wh	0	0	0	0	0	-	-	-
UMICH 17	No KP.2 MV	36	M	Wh	5	3	1	1	0	1208	368	WT-P/WT-P/WT-P/BA.5-P/XBB.1.5-P
UMICH 18	No KP.2 MV	23	M	As	3	3	0	0	0	90	1033	WT-P/WT-P/WT-P
UMICH 19	No KP.2 MV	33	F	As	5	3	1	1	0	271	383	WT-P/WT-P/WT-P/BA.5-P/XBB.1.5-P
UMICH 20	No KP.2 MV	20	F	Wh	3	1	1	1	0	59	364	WT-P/BA.5-M/XBB.1.5-M
UMICH 21	No KP.2 MV	61	M	Wh	5	3	1	1	0	109	402	WT-P/WT-P/WT-P/BA.5-P/XBB.1.5-P
UMICH 22	No KP.2 MV	48	F	Af	4	3	0	1	0	109	414	WT-P/WT-P/WT-P/XBB.1.5-P
UMICH 23	No KP.2 MV	64	F	Wh	2	2	0	0	0	-	1092	WT-M/WT-M
UMICH 24	No KP.2 MV	35	F	Wh	3	3	0	0	0	1427	1163	WT-P/WT-P/WT-P
UMICH 25	No KP.2 MV	61	M	Wh	4	3	1	0	0	972	789	WT-M/WT-M/WT-M/BA.5-P
UMICH 26	No KP.2 MV	42	F	As	5	3	1	1	0	155	429	WT-P/WT-P/WT-P/BA.5-P/XBB.1.5-P
UMICH 27	No KP.2 MV	42	F	Af	0	0	0	0	0	507	-	-
UMICH 28	No KP.2 MV	50	F	Wh	3	3	0	0	0	37	1164	WT-P/WT-P/WT-P
UMICH 29	No KP.2 MV	44	F	Other	4	2	1	1	0	88	515	WT-U/WT-P/BA.5-P/XBB.1.5-M
UMICH 30	No KP.2 MV	36	F	Wh	0	0	0	0	0	Unknown	-	-
UMICH 31	No KP.2 MV	53	M	Wh	5	3	1	1	0	235	552	WT-P/WT-P/WT-P/BA.5-P/XBB.1.5-P
